# Transcriptional control of the F_0_F_1_-ATP synthase operon of *Corynebacterium glutamicum*: SigmaH factor binds to its promoter and regulates its expression at different pH values

**DOI:** 10.1111/1751-7915.12022

**Published:** 2013-01-09

**Authors:** Mónica Barriuso-Iglesias, Carlos Barreiro, Alberto Sola-Landa, Juan F Martín

**Affiliations:** 1 Instituto de Biotecnología de León (INBIOTEC), Parque Científico de LeónAv. Real, 1, 24006, León, Spain; 2 Departamento de Biología Molecular de la Universidad de León, Facultad de Ciencias Biológicas y Ambientales, Campus de Vegazanas/n, 24071, León, Spain

## Abstract

*Corynebacterium glutamicum* used in the amino acid fermentation industries is an alkaliphilic microorganism. Its *F*_0_F_1_-ATPase operon (*atpBEFHAGDC*) is expressed optimally at pH 9.0 forming a polycistronic (7.5 kb) and a monocistronic (1.2 kb) transcripts both starting upstream of the *atpB* gene. Expression of this operon is controlled by the SigmaH factor. The sigmaH gene (*sigH*) was cloned and shown to be co-transcribed with a small gene, *cg0877*, encoding a putative anti-sigma factor. A mutant deleted in the *sigH* gene expressed the *atpBEFHAGDC* operon optimally at pH 7.0 at difference of the wild-type strain (optimal expression at pH 9.0). These results suggested that the SigmaH factor is involved in pH control of expression of the *F*_0_F_1_ ATPase operon. The SigmaH protein was expressed in *Escherichia coli* fused to the GST (glutathione-S-transferase) and purified to homogeneity by affinity chromatography on a GSTrap HP column. The fused protein was identified by immunodetection with anti-GST antibodies. DNA-binding studies by electrophoretic mobility shift assays showed that the SigH protein binds to a region of the *atpB* promoter containing the sigmaH recognition sequence (−35)TTGGAT…18nt…GTTA(−10). SigmaH plays an important role in the cascade of control of pH stress in *Corynebacterium*.

## Introduction

*Corynebacterium glutamicum* is a Gram-positive coryneform actinobacteria widely used in biotechnology for industrial production of amino acids and amino acid-derived metabolites (Schaffer and Burkovski, 2005). Its molecular genetics has received increasing attention in the last two decades (Correia *et al*., 1994; Martín and Gil, 1999) and the genome of four strains of *C. glutamicum* ATCC 13032 has been sequenced by different research groups (Nakagawa, 2002; Kalinowski *et al*., 2003; Lv *et al*., 2011; 2012).

*Corynebacterium glutamicum* is usually grown at neutral pH, but, in a previous study, we found that it is an alkaliphilic bacteria with an optimal pH of 9.0 (Barriuso-Iglesias *et al*., 2006). The *F*_0_F_1_-ATPase operon of this microorganism that includes the genes *atpBEFHAGDC* is overexpressed at pH 9.0 as compared with pH 7.0 (Barriuso-Iglesias *et al*., 2006; 2008). However, the molecular mechanism(s) by which *C. glutamicum* controls the expression of this important operon encoding all components of the membrane ATPase remains unknown.

The transcriptional regulators of *C. glutamicum* include several types of proteins (Brinkrolf *et al*., 2007). Sigma factors play a very important role in triggering cascades of gene expression. These cascades have been studied in *Bacillus subtilis* and other Gram-positive bacteria. The number of sigma factors encoded in the genomes of microorganisms is related to the complexity of its metabolism. Mycoplasma contains just one sigma factor (Fraser *et al*., 1995; Himmelreich *et al*., 1996), whereas the soil actinomycete *Streptomyces coelicolor* which undergoes complex biochemical changes and differentiation processes has been reported to contain 65 sigma factor (or sigma-factor-like) genes (Bentley *et al*., 2002). In the *C. glutamicum* genome there are seven different genes encoding sigma factors. Two of them SigB and SigA, appear to be sigma factors for the housekeeping vegetative genes (Oguiza *et al*., 1997). Five other SigC, SigD, SigE, SigH and SigM belong to the so-called ECF (extracytoplasmic function) family (Engels *et al*., 2004). *SigH* has been reported to play a role in the expression of genes involved in heat-shock and oxidative stress but its role in the control of other genes or operons remains obscure (see *Discussion*).

Initial studies in our group suggested that the *F*_0_F_1_-ATPase operon may be under control of the SigmaH (SigH) factor (Barriuso-Iglesias *et al*., 2006). It was, therefore, important to study the expression of the *F*_0_F_1_-ATPase operon in a *C. glutamicum* mutant defective in *sigH* as compared with its response to pH changes in the wild-type parental strain. Furthermore, it was also important to analyse the possible SigH binding to the *atpBEFHAGDC* promoter in this microorganism.

## Results

### Deletion of the *sigH* gene causes a drastic change in the pH response of the *F*_0_F_1_-ATPase operon

In the *C. glutamicum* genome, the *sigH* gene (621 nt, access No. BX 927147.1, Kalinowski *et al*., 2003) is preceded upstream by an open reading frame (ORF) encoding a hypothetical protein, and is followed in the same orientation by a small gene encoding a putative anti-sigma factor (*cg0877*), also named *rsrA* o *rshA*. The *sigH* and *cg0877* genes are separated by only three nucleotides. Downstream of *cg0877* and separated by 232 bp in the complementary strand, is located the *whiB1* gene (also named *whcE*, Kim *et al*., 2005b) that encodes a transcriptional factor.

As reported previously, *C. glutamicum* is an alkaliphilic microorganism and the *F*_0_F_1_-ATPase operon is expressed optimally at pH 9.0 (Barriuso-Iglesias *et al*., 2006). Since a putative SigH binding site was found in the promoter of the *atpBEFHAGDC* operon encoding the *F*_0_F_1_-ATPase complex, we decided to compare expression of this operon in the wild-type *C. glutamicum* ATCC 13032 and in a Δ*sigH* mutant derived from it (Engels *et al*., 2004).

**Figure 1:**
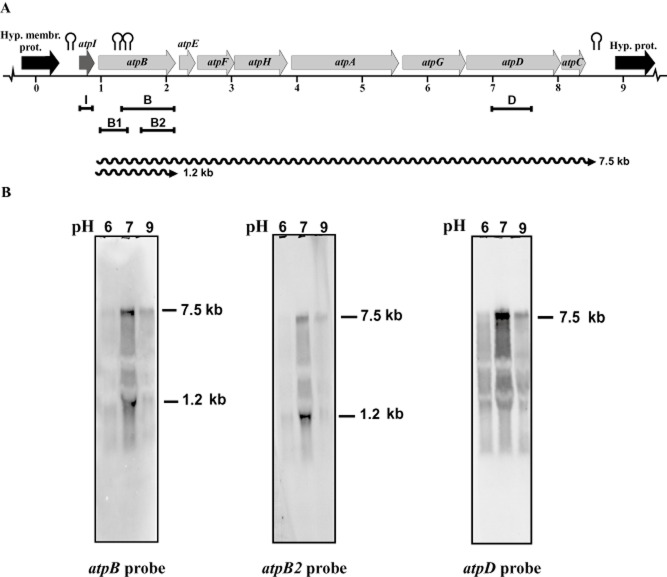
Transcription of the *F*_0_F_1_-ATP synthase operon in *C. glutamicum* Δ*sigH* at different pH conditions.A. Scheme of the *f0f1* operon. The probes B, B1, B2 and D are indicated by black solid bars. Wavy lines represent the transcripts of the operon. Hairpin (stem and loop) symbols represent putative transcription terminators (see Barriuso-Iglesias *et al*., 2006).B. Northern hybridizations of the *F*_0_F_1_-ATP synthase operon with probes B, B2 and D. The sizes of the hybridizing bands are indicated on the right of the panels. [Correction added on 28 January 2013 after first online publication: Figure 1 and its legend have been modified to remove the lower panels (16S probes).]

Northern analysis of the transcripts formed from the *atpBEFHAGDC* operon in the wild-type strain were made using the probes named B, B1, B2 and D, internal to *atpB* (probes B, B1 and B2) and *atpD* genes respectively. The size of the transcripts (7.5 and 1.2 kb) was the same in the wild type and the Δ*sigH* mutant, but the pattern of response to pH was clearly different (Fig. 1). The best results were obtained with probes B, B2 and D. Using probes B or B2 we observed two transcripts of 7.5 and 1.2 kb respectively, as described for the wild-type (Barriuso-Iglesias *et al*., 2006). With probe D, only the 7.5 kb transcript was found. The large 7.5 kb transcript corresponds to a polycistronic transcript of the *atpBEFHAGDC* operon, whereas the short 1.2 kb transcript obtained with probes B or B_2_, but not with probe D, corresponds to a monocistronic mRNA of the first gene of the operon.

Interestingly, whereas maximal expression of the *F*_0_F_1_-ATPase operon in the wild-type strain was observed at pH 9.0 with any of the three probes (Barriuso-Iglesias *et al*., 2006), optimal expression of this operon in the Δ*sigH* mutant occurred at pH 7.0 (Fig. 1). These results suggest that expression of the *F*_0_F_1_-ATPase operon is under the control of the ECF σ^H^ factor.

### Transcriptional organization of the *sigH-cg0877* region

**Figure 2:**
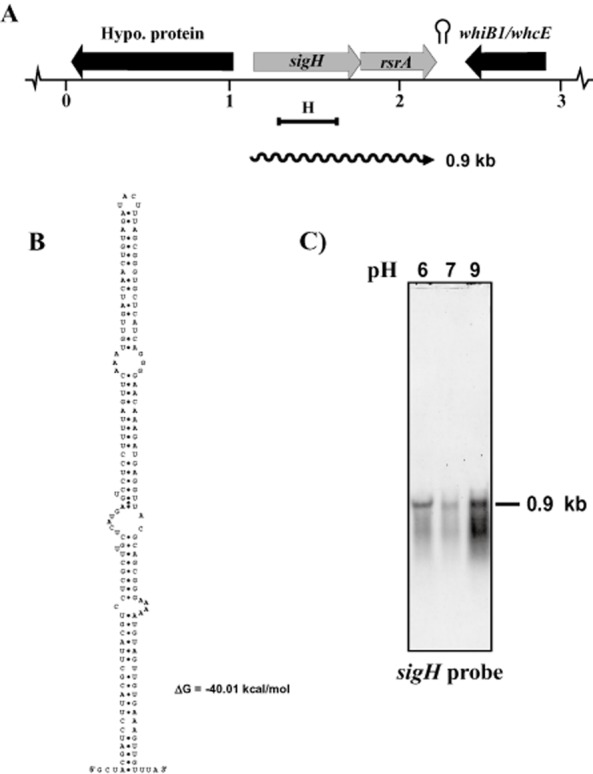
Transcriptional analysis of the *sigH* gene in *C. glutamicum* ATCC 13032 at different pH conditions.A. Organization scheme of the *sigH* region in *C. glutamicum* ATCC 13032. The probe is shown by a black solid bar. The *sigH-rsrA* transcript (0.9 kb) is indicated by a wavy line. The hairpin (stem and loop) symbol represents a putative transcription terminator.B. Stem and loop structure of the putative transcriptional terminator found downstream of *rsrA*. The free energy (ΔG) is indicated.C. Northern analysis of the *sigH-rsrA* operon with probe H. The size of the hybridizing band is indicated on the right of the panel. [Correction added on 28 January 2013 after first online publication: Figure 2 and its legend have been modified to remove the lower panels (16S probes).]

SigH is encoded by a 621 nt ORF and is very closely linked to *cg0877* (3 nt separation). In order to study the transcriptional response of *sigH* to pH stress, we analysed the transcription of *sigH* using a sensitive RNA–RNA hybridization procedure with an antisense RNA probe (see *Experimental procedures*). The 615 nt RNA probe obtained was named probe H (Fig. 2A).

Hybridizations with total RNA of the wild-type *C. glutamicum* grown at pH 6.0, 7.0 and 9.0 using the same amount of total RNA showed a single 0.9 kb transcript that appears to correspond to the bi-cistronic transcript of *sigH* and *cg0877* [Correction added on 28 January 2013 after first online publication: the text in parenthesis referring to 16S rRNA probe has been deleted from the above statement]. The expression of these genes was clearly higher at alkaline pH (9.0) than at lower pH 7.0 (Fig. 2C).

The possible joint transcription of *sigH* and *cg0877* (encoding a putative anti-sigma factor) was supported by the lack of an identifiable separate promoter for *cg0877* and by the finding of a putative strong transcriptional terminator downstream of *cg0877* formed by an inverted repeat sequence of 72 nt, with a calculated ΔG of −40 kcal mol^−1^ (Fig. 2B). This putative transcriptional terminator belongs to the type I, or *Mycobacterium* type (Unniraman *et al*., 2002), and is located 87 nt downstream of *cg0877* stop codon (see *Discussion*).

### Identification of the SigH-encoded protein

**Figure 3:**
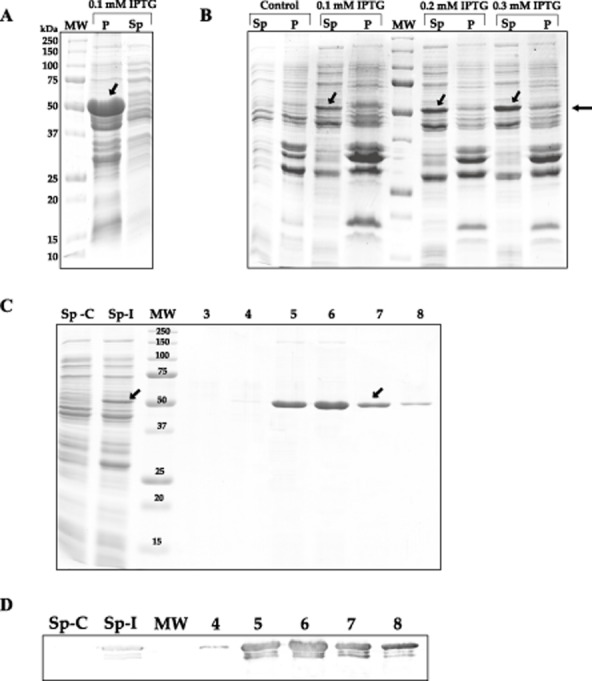
Overexpression of SigH as a GST–SigH fused protein in *E. coli*.A. SDS-PAGE gel of the GST–SigH overexpression at 37°C.B. SDS-PAGE gel of the GST–SigH overexpression at 25°C and different IPTG concentrations. Control: pGEX–SigH without induction; Sp: supernatant (soluble fraction); P: pellet (insoluble fraction). Molecular size markers, in kDa, are indicated on the left side of panel A. Arrows show the GST–SigH fusion protein overexpression.C. Purification of the GST–SigH fusion protein by affinity chromatography on Glutathione Sepharose. Samples were analysed in 12.5% acrylamide SDS-PAGE gels. Sp-C/Sp-I: Control or induced supernatant. MW: Molecular size markers, in kDa. 3–8: Elution fractions from the affinity chromatography. Arrows show the GST–SigH fusion protein overexpressed and purified.D. Inmunodetection of the GST–SigH fusion protein by Western blot analysis with anti-GST antibodies. Sp-C/Sp-I: 20 μg of non-induced and induced supernatants. MW: Molecular size markers (in kDa). 4–8: 5 μl of the affinity chromatography eluted fractions.

A very high expression of *sigH* was obtained in *Escherichia coli* cultures at 37°C following addition of IPTG (0.1 mM). However, under these conditions most of the SigH protein was insoluble (inclusion bodies, Fig. 3A). The best condition to obtain soluble SigH protein was growing the culture at 25°C before and after induction with a 0.3 mM IPTG concentration (Fig. 3B). A band of the expected size (49 kDa) for the GST–SigH protein was observed in extracts of the induced cultures as compared with the non-induced ones (Fig. 3C). The identity of the induced protein was confirmed by inmunodetection with anti-GST antibodies (Fig. 3D).

The fused GST–SigH protein was purified from the soluble fraction of *E. coli* transformants containing the pGEX–SigH construction using GST-Trap HP colums, as described in *Experimental procedures*. After washing with 15 ml of PBS buffer, the GST–SigH protein was eluted with 10 mM reduced glutathione in 50 mM Tris-HCl buffer pH 8.0. The fractions were collected, analysed by SDS-PAGE and tested with antibodies against GST. As shown in Fig. 3D, almost all of GST–SigH protein (reactive with anti-GST antibodies) was found in fractions 5–8 of the 20 collected fractions. In this way, about 500 μg of pure GST–SigH protein was obtained per 100 ml of *E. coli* culture. The nature of the purified protein was identified by peptide mass fingerprinting as described in *Experimental procedures*.

### The purified GST–SigH binds to the *atpBEFHAGDC* operon promoter

Binding of the purified GST–SigH protein to the promoter of the *atpBEFHAGDC* operon was tested by the electrophoretic mobility shift assay (EMSA). For this purpose, the *f0f1* operon promoter was cloned as a 357 bp DNA fragment using a high fidelity DNA polymerase (*Platinum® Pfx*, Invitrogen) and oligonucleotides PxB-U and PxB-D2 as primers (Table 1). The amplified DNA fragment was purified by filtration through *GFX PCR DNA and gel band purification kit* (GE Healthcare) and cloned in pGEM®-T Easy (this construction was named pGEM-P*f0f1*).

**Table 1:** Strains, plasmids and oligonucleotides used in this work

Bacterial strains/plasmids/oligonucleotides	Relevant characteristics	Source/reference
Bacterial strain		
*E. coli* DH5α	φ80d*lac*ZΔM15, *recA*1, *endA*1, *gyr*AB, *thi*-1, *hsdR*17(r_K_−, m_K_+), *supE*44, *relA*1, *deo*R, Δ*(lacZYA*-*arg*F) U169, *pho*A	Hanahan (1983)
* C. glutamicum* ATCC 13032	Wild type	American Type Culture Collection
* C. glutamicum* Δ*sigH*	In frame deletion of the *sigH* gene	Abe *et al*. (1967)
		Engels *et al*. (2004)
Plasmid		
pGEM®-T Easy Vector	Amp^R^, cloning vector	Promega Corporation, Madison, USA.
pGEM-TB	pGEM®-T Easy + 750 bp internal fragment of *atpB*	Barriuso-Iglesias *et al*. (2006)
pGEM-TB1	pGEM®-T Easy + 275 bp internal fragment of *atpB*	Barriuso-Iglesias *et al*. (2006)
pGEM-TB2	pGEM®-T Easy + 350 bp internal fragment of *atpB*	Barriuso-Iglesias *et al*. (2006)
pGEM-TD	pGEM®-T Easy + 850 bp internal fragment of *atpD*	Barriuso-Iglesias *et al*. (2006)
pGEM-TH	pGEM®-T Easy + 615 bp internal fragment of *sigH*	This work
pGEM-P*f0f1*	pGEM®-T Easy + Promoter region of F_0_F_1_ operon	This work
pGEX-2T	Vector system for protein expression	GE Healthcare
pGEM-TSigH	pGEM®-T Easy + *C.* *glutamicum sigH* gene	This work
pGEX–SigH	pGEX-2T + *C.* *glutamicum sigH* gene	This work
Oligonucleotides used as primers		
SIGH-U:	5′-GAAAACCGAACCGGCACA-3′	
SIGH-D:	5′-CTCCGAATTTTTCTTCATGT-3′	
Forward:	5′-GTAAACGACGGCCAGT-3′	
Reverse:	5′-GGAAACAGCTATGACCATG-3′	
16S-3:	5′-GGCCCCCGTCAATTCCTTT-3′	
16S-5:	5′-GGCGGCGTGCTTAACACAT-3′	
EXH-U:	5′-AAAGGATCCCACATGGCTGA-3′	
EXH-D:	5′-GTTGAGAATTCGTCATCGTT-3′	
5′-pGEX:	5′-GGGCTGGCAAGCCACGTTTGGTG-3′	
3′-pGEX:	5′-CCGGGAGCTGCATGTGTCAGAGG-3′	
PxB-U:	5′-AGTGGATCCGTGCTGGGAAA-3′	
PxB-D2:	5′-CGGAGCTCCGTCGCACATC-3′	

Promoter DNA-binding assays were performed with the purified GST–SigH protein in buffers A and B, differing essentially in the magnesium salt utilized and in the presence of polyethylene glycol (PEG) in buffer B.

**Figure 4:**
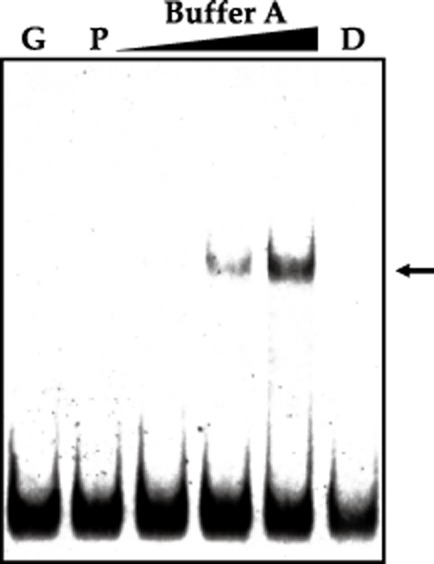
Electrophoretic mobility-shift assays of the *f0f1* promoter region using increasing concentrations (25, 50 and 100 pmol) of the GST–SigH protein. The assays were performed in buffer A as indicated in *Experimental procedures*. Lane G, control with pure GST protein (150 pmol). Lane P, probe without protein. Lane D, reaction diluted with excess (500-fold) unlabelled probe. Note the formation of one DNA–protein complex (arrow) at increasing protein concentration.

Results of the binding experiments are shown in Fig. 4. It is observed that there is a good binding of the GST–SigH protein, but not of the separate GST protein used as control, to the labelled *F*_0_F_1_-ATPase promoter, in buffer A; however, binding was barely visible in buffer B (not shown), indicating that some of the components of this buffer (most likely PEG) are unfavourable for the protein-DNA interaction. The intensity of the retarded band was proportional to the amount of GST–SigH protein used (25–100 pmol) and the labelled band was not formed when the reaction was supplemented with additional unlabelled probe. These results show that SigH binds to the promoter of the *atpBEFHAGDC* operon and, therefore, participates in the pH control of this operon in *C. glutamicum*. The used DNA fragment contains the consensus sequence GGAT…18nt…GTTA in the −35 to −10 region of the promoter.

## Discussion

Sigma factors are very important auxiliary components of the RNA polymerase since they determine the choice of promoters and, therefore, the genes to be expressed under specific physiological conditions. A survey of the sigma promoters in 19 corynebacterial species has been made (Pátek and Nešvera, 2011). The σ^A^ housekeeping genes sigma factor, which belongs to the σ^70^ class, is similar to the σ^A^ of *E. coli* and *Mycobacterium tuberculosis* (Oguiza *et al*., 1996). The alternative sigma factors σ^B^, σ^E^, σ^H^ and σ^M^ are present in different corynebacteria and appear to be involved in expression of genes for several important processes. Thus, σ^B^ is involved in the response to several stress conditions and in the expression of genes required for the transition of the exponential growth phase to the stationary phase (Halgasova *et al*., 2002; Larisch *et al*., 2007; Ehira *et al*., 2008). The σ^E^ factor (for the so-called extracytoplasmic factors) has a role on the expression of genes in response to the cell-wall damage (Pátek *et al*., 2003). The σ^H^ is involved in the response to heat stress (Barreiro *et al*., 2009; Ehira *et al*., 2009; Busche *et al*., 2012). σ^M^ controls the expression of a few genes involved in the control of disulfide stress (Nakunst *et al*., 2007).

It is noteworthy that *sigH* is involved in the control of the expression of the genes encoding two other sigma factors, namely σ^M^ (Nakunst *et al*., 2007) and σ^B^ (Ehira *et al*., 2008), in addition to be required for the expression of its own gen (Kim *et al*., 2005a).

One of the best known SigH-dependent systems is that of *B. subtilis* that may serve as a model for other Gram-positive bacteria. In *B. subtilis*, SigH (encoded by the *spo0H* gene) is the first in a cascade of five sigma factors that lead to reprogramming the bacterial metabolism to form heat-resistant endospores (Carter and Moran, 1986; Dubnau *et al*., 1988). After entering the stationary phase, following derepression of *spo0H* (encoding the SigH factor) several proteins involved in sporulation and development of competence are formed (Grossman, 1995; Fujita and Sadaie, 1998).

It is interesting that in *B. subtilis* cultures grown at low pH values, the genes regulated by Spo0H (including the own *spo0H*) are expressed only at basal levels, whereas increased levels of Spo0H (SigH) are formed following addition of compounds that maintain higher pH values (Cosby and Zuber, 1997). The increased levels of Spo0H lead to high expression of *spo0A*, a key regulator of the late differentiation steps. Spo0A in its phosphorylated form represses formation of AhrB, which controls negatively the transcription of *spo0H*, thus feedback regulating the levels of Spo0H. This circuit is extremely important in the control of sporulation and its relation with pH changes.

The role of *sigH* has been also studied in some detail in *Mycobacterium tuberculosis*, where it is induced by heat-shock or by treatment with diamines (Graham and Clark-Curtiss, 1999; Raman *et al*., 2001). Under reducing conditions in the cell, the expression of *sigH* is regulated negatively by an anti-sigma factor protein named RshA (Song *et al*., 2003) by protein–protein interaction. Using a *M. tuberculosis sigH* null mutant (Manganelli *et al*., 2002) it was observed that several other transcriptional factors are under control of SigH including SigB and SigH itself, in addition to enzymes involved in redox metabolism (thioredoxin and thioredoxin–oxidoreductase) and enzymes of cysteine and molybdopterin biosynthesis and the chaperones Hsp70 and ClpB.

In *S. coelicolor*, the *sigH* gene is linked to the *ushX* gene encoding an anti-sigma factor. Both genes are co-transcribed. As in *M. tuberculosis*, the SigH–UshX complex, in response to external stress signals, dissociates releasing the sigma factor that binds to the RNA polymerase and recognizes the sigH-dependent promoters (Kormanec *et al*., 2000; Sevcikova and Kormanec, 2002; Kormanec and Sevcikova, 2002a,b).

Much less is known about the role of SigH in corynebacteria. Five of the seven putative sigma factors in the genome of *C. glutamicum*, SigC, SigD, SigE, SigH and SigM belong to the ECF subclass (Engels *et al*., 2004). *sigH* of *C. glutamicum* encodes a small protein of 206 amino acids that is closely linked to a downstream ORF (*cg0877*) separated by only 3 bp that encodes a putative anti-sigma factor (named RsrA) that may play a role similar to UshX in *S. coelicolor*. Interestingly, downstream of *sigH*-*rsrA* in opposite orientation and separated by a putative strong transcriptional terminator (Fig. 2A and B) is located the *whiB1* gene, also named *whcE*, that encodes another putative transcriptional factor (Kim *et al*., 2005b).

As described in this work, purified SigH protein binds to the promoter of the *atpBEFHAGDC* operon. Expression of the *sigH* gene responds to the pH of the culture, being clearly higher at pH 9.0 than at neutral or slightly acid pH values. In this way, SigH mediates pH control of the *F*_0_F_1_-ATPase operon in *C. glutamicum*, which was previously reported to be optimal at about pH 9.0 (Barriuso-Iglesias *et al*., 2006). A *sigH* null mutant shows optimal expression of the *atp* operon at neutral pH in contrast to the wild-type strain. SigH has been reported to modulate its own expression in *C. glutamicum* that is optimal in the stationary phase of growth (Kim *et al*., 2005a).

SigH may participate in the control of cellular processes through other sigma factors. Halgasova and colleagues (2002) reported that in *C. glutamicum* subsp. *flavum*, SigH regulates the expression of *sigB* under situations of heat-, cold- or acid-stress. This observation correlates well with our own observation of SigH response to acid or basic pH values.

In addition to the control of the *F*_0_F_1_-ATPase operon, SigH has been reported to play an important role in the control of several types of stress in *C. glutamicum*. We observed that SigH regulates in a positive manner the expression of the *dnaK* operon that encodes a chaperone involved in heat-shock (Barreiro *et al*., 2004) and the expression of the heat-shock regulators HspR and ClgR under conditions of severe heat-stress (Barreiro *et al*., 2004; 2009; Engels *et al*., 2004; Ehira *et al*., 2009). SigH also regulates the expression of *whcE*, the regulator of the thioredoxin system (Kim *et al*., 2005b) and the *clpC* and *clpP1P2* that encode heat-shock proteases (Engels *et al*., 2004).

**Figure 5:**
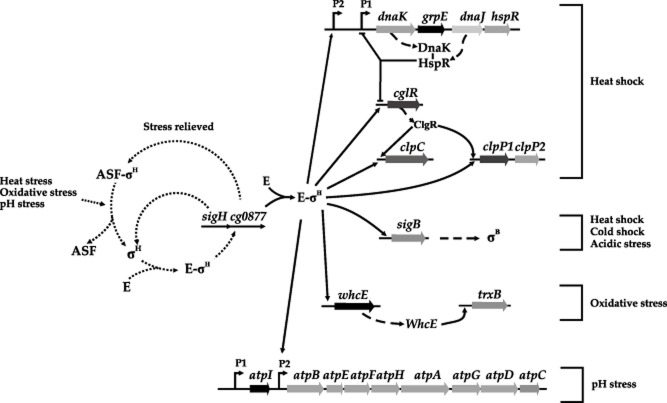
Proposed SigmaH factor (σ^H^) regulatory network in *C. glutamicum* ATCC 13032. Solid line arrows indicate induction whereas blunt-ended solid lines indicate repression. Dotted lines represent regulations known to occur in other Gram-positive bacteria. Dashed lines with arrowheads indicate translation (formation) of certain proteins. ASF, anti-sigma factor; P, promoter.

In summary, SigH exerts the upper level of control of genes involved in the stress response to unfavourable nutritional or environmental conditions. Up to 47 regulatory interactions have been described to be mediated by SigH (Baumbach *et al*., 2006; 2007). A summary of those interactions is shown in Fig. 5 taking into account the results presented in this article, and those reported by other authors (Halgasova *et al*., 2002; Barreiro *et al*., 2004; Engels *et al*., 2004; Kim *et al*., 2005a,b; Barriuso-Iglesias *et al*., 2006).

## Experimental procedures

### Bacterial strains, plasmids, and culture conditions

The bacterial strains, plasmids and oligonucleotides used in this work are listed in Table 1. *Corynebacterium glutamicum* Δ*sigH* was kindly provided by Jens Schweitzer and Michael Bott (Jülich, Germany). *Escherichia coli* was grown in Luria–Bertani (LB) broth (Sambrook and Russell, 2001) at 37°C. *Corynebacterium glutamicum* strains were grown in trypticase soy broth (TSB) at 30°C and at different pH conditions, in flasks. *Escherichia coli* transformants were selected in presence of ampicillin (100 μg ml^−1^), kanamycin (50 μg ml^−1^) or chloramphenicol (25 μg ml^−1^). *Corynebacterium glutamicum* transformants were selected on media with kanamycin (30 μg ml^−1^) or chloramphenicol (12 μg ml^−1^).

### DNA isolation and manipulation

*Escherichia coli* plasmid DNA was obtained by alkaline lysis. Total *C. glutamicum* DNA was prepared as described by Martín and Gil (1999). DNA manipulations were performed as reported by Sambrook and Russell (2001). DNA fragments were isolated from agarose gels using the *GFX PCR DNA and Gel Band Purification Kit* (GE Healthcare). *Escherichia coli* cells were transformed by standard methods (Sambrook and Russell, 2001), whereas *C. glutamicum* cells were transformed by electroporation (van der Rest *et al*., 1999).

### RNA extraction

Total RNA from corynebacteria grown at different pHs (OD_600_ 3.5–4.0) was extracted essentially as described by Eikmanns and colleagues (1994), except that the cell pellet obtained after centrifugation was frozen with liquid nitrogen and kept at −70°C until RNA extraction (Barreiro *et al*., 2001). The RNA concentration was determined spectrophotometrically by the absorbance at 260 nm.

### Northern hybridizations

Denaturing RNA electrophoresis was performed in 0.9% agarose gels in MOPS buffer (20 mM MOPS, 5 mM sodium acetate; 1 mM EDTA pH 7.0) with 17% (v/v) formaldehyde. RNA (30–40 μg) was dissolved in denaturing buffer {50% formamide, 20% formaldehyde, 20% MOPS (5×) with 10% DYE [50% glycerol, 0.25% (w/v) bromophenol blue, 0.25% (w/v) xylene cyanol, 1 mM EDTA] (Sambrook and Russell, 2001) and 1% ethidium bromide}. RNA probes for *atpI*, *atpB*, *atpD* and *sigH* were labelled with digoxigenin and Northern hybridizations were performed following the instructions of the *DIG Northern Starter Kit* manufacturer (Roche Applied Science). The hybridization temperature was 68°C.

### Preparation of RNA probes

For transcriptional analysis of the *sigH* gene a RNA probe was prepared as follows: a 615 bp fragment internal to *sigH* was cloned by PCR in pGEM®-T Easy. The *sigH* fragment was excised with the *ApaI* endonuclease and placed under control of the SP6 RNA polymerase promoter in vector pGEM®-T Easy.

### Protein expression and purification

To study the possible binding of the SigH protein to the promoter region of the *atpBEFHAGDC* operon, it was necessary to obtain an active sigmaH protein. The *sigH* gene was overexpressed in *E. coli* using the pGEX-2T expression system (GE Healthcare). *sigH* was amplified as a 660 bp PCR fragment from total DNA of *C. glutamicum* with *Platinum*® *pfx* DNA polymerase, using the ExH-U/ExH-D primers (Table 1), and cloned into pGEM®-T Easy vector (Promega). The insert was excised by digestion with *Bam*HI and *Eco*RI (the restriction sites were in the upstream and downstream primers respectively) and was cloned into pGEX-2T expression plasmid (GE Healthcare). The new pGEX–SigH plasmid was introduced in *E. coli* DH5α. The correct integration of the insert was established by sequencing. To obtain the fusion protein, *E. coli* cells were grown in LB medium in an orbital shaker (250 r.p.m.) to an OD_600_ of 0.6 and under the four following conditions: (i) culture growth at 37°C before and after IPTG induction, (ii) growth at 25°C prior to induction and at 37°C following IPTG addition, (iii) growth at 25°C prior to induction and at 30°C following IPTG addition and (iv) growth at 25°C before and after IPTG addition. These four conditions were combined with increasing IPTG concentrations from 0.1 to 0.3 mM.

Cells were harvested by centrifugation, washed twice with NaCl 0.9% and lysed by sonication. The soluble fraction was separated by centrifugation and the protein was purified in an ÄKTA-FPLC using a Glutathione Sepharose 4B column (GE Healthcare). The protein was eluted with 10 mM reduced glutathione (in 50 mM Tris-HCl, pH 8.0) following the manufacturer's recommendations and conserved in 40% glycerol at −80°C before use. Protein concentrations were determined with the Bradford reagent (Bio-Rad).

### Peptide mass fingerprint

The protein band of interest were manually excised from stained gels, placed in an Eppendorf tube and washed twice with double distilled water. The proteins were digested following the method of Havlis and colleagues (2003) and processed for further analysis as described previously (Jami *et al*., 2010). The samples were analysed with a 4800 Proteomics Analyzer matrix-assisted laser desorption ionization time-of-flight (MALDI-TOF/TOF) mass spectrometer (AB Sciex). All MS spectra were internally calibrated using peptides from the trypsin auto digestion. The analysis by MALDI-TOF/TOF mass spectrometry produced peptide mass fingerprints, and the peptides observed were collected and represented as a list of monoisotopic molecular weights with a signal-to-noise (S/N) ratio greater than 20 using the 4000 Series Explorer v3.5.3 software (AB Sciex). For protein identification, Mascot generic files were automatically created and used to interrogate a non-redundant protein database (NCBInr) using a local license of Mascot v 2.2.04 from Matrix Science through the Global Protein Server v 3.6 (AB Sciex).

### Electrophoretic mobility shift assays (EMSA)

DNA-binding tests were performed by EMSA. The DNA fragments were labelled at both ends with digoxigenin using the *DIG Oligonucleotide 3′-End Labelling Kit*, 2nd Generation (Roche Applied Science). The binding reaction was performed in two reaction buffers: buffer A) 10 mM Tris-HCl pH 8.0, 0.4 mM MgCl_2_, 10 mM KCl, 0.2 mM DTT, 1.6 mM glutathione and 0.01% Nonidet P40 (Sola-Landa *et al*., 2002); buffer B) 25 mM Tris-acetate pH 8.0; 8 mM Mg-acetate; 10 mM KCl; 1 mM DTT and 3.5% w/v PEG 6000 (Wigneshweraraj *et al*., 2002). The binding reaction also contained 50 μg ml^−1^ poly[d(I-C)]. The samples were loaded onto a 5% polyacrylamide (29:1) native gel in 0.5× TBE buffer. After electrophoresis (5 h, 80 V), DNA was electroblotted onto a nylon membrane in 0.5× TBE buffer (1 h, 200 mA). The DNA was fixed by UV cross-linking, detected with anti-digoxigenin antibodies and developed by chemiluminiscence with the CDP-Star TM reagent (Roche Applied Science).
